# Evaluation of the Loading, Unloading, and Permanent Deformation of Newly Available Epoxy Resin Coated Ni-Ti Wires Using Self-Ligating Brackets

**DOI:** 10.1155/2017/8085067

**Published:** 2017-05-23

**Authors:** Hazel Garro-Piña, María Cristina Jiménez-Cervantes, Ricardo Ondarza-Rovira, Roberto Justus, Salvador García-López

**Affiliations:** ^1^Private Practice, San José, Costa Rica; ^2^Department of Orthodontics, Universidad Intercontinental, Mexico City, Mexico; ^3^Instituto Nacional de Investigaciones Nucleares and Universidad Intercontinental, Mexico City, Mexico; ^4^Universidad Intercontinental, Mexico City, Mexico; ^5^Universidad Intercontinental e Universidad Autónoma Metropolitana, Mexico City, Mexico

## Abstract

**Aim:**

The purpose of this study was to evaluate the load and unload deflection and permanent deformation of round 0.016′′ and rectangular 0.016′′ × 0.022′′ regular and coated Ni-Ti wires.

**Materials and Methods:**

Ni-Ti archwires produced by two manufacturers were evaluated. Both regular and coated round and rectangular Ni-Ti wire segments (*n* = 15) from each group were submitted to a three-point bending test. Both types of wires were evaluated for permanent deformation at the end of a recovery cycle.

**Results:**

The coated round 0.016′′ Ni-Ti wires produced a significantly lower force in loading (*p* < 0.01) and unloading (*p* < 0.01) than regular wires of the same manufacturer and size. There was no significant difference in permanent deformation between coated and regular round Ni-Ti wires from the same company. For rectangular 0.016 × 0.022′′ Ni-Ti wires, there was a significant difference in the loading evaluation, but the unloading test presented no significant differences. The permanent deformation of the rectangular wires revealed no significant difference between them.

**Conclusion:**

The addition of an esthetic coating to these new Ni-Ti wires produced changes in their mechanical properties, manifested as a reduction in the applied force, which should be considered in clinical management.

## 1. Introduction

Orthodontic treatment in adults has begun to seek more esthetic orthodontic appliances. Although clear brackets have been used worldwide for a long time [1], the archwires continue to be made of metals, such as stainless steel, nickel titanium, or titanium molybdenum alloy [2]. Fiber-reinforced and coated metallic wires have been developed to promote a more appealing appearance during orthodontic treatment [3]. The emerging coated metallic archwires include tooth-colored polymer made of polytetrafluoroethylene or epoxy resin, which is heat treated in a chamber furnace [4]. This approach improves the esthetics but may alter the mechanical properties of the wires. Some studies have shown that the color tends to change with time and that the coated wire splits during mechanics, exposing the underlying metal [5, 6]. Although the coating reduces friction with the bracket [7], further increased surface roughness can increase the friction coefficient [8]; therefore, coating stability is needed in this type of wire to maintain a force comparable to that of most regular Ni-Ti archwires. The mechanical properties of these types of wires can be assessed using 3-point bending tests, which evaluate the load and unload force deflection in the horizontal or vertical direction, allowing one to determine the biological nature of tooth movement [9]. However, although coated Ni-Ti archwires present certain disadvantages, this kind of wire continues to be marketed; consequently, new manufacturers are available today. No studies have yet been conducted to evaluate the mechanical properties of these new esthetic coated fabricated archwires and to compare them with the properties of conventional Ni-Ti wires of the same dimensions from the same manufacturer. The purpose of this study was to evaluate and compare the force levels and permanent deflection of round 0.016′′ and rectangular 0.016′′ × 0.022′′ regular and coated Ni-Ti wires from three different companies using self-ligating brackets under the same testing conditions.

## 2. Materials and Methods

Coated and regular 0.016′′ round and rectangular 0.016′′ × 0.022′′ Ni-Ti wires from three different manufacturers (Ah Kim Pech, 03100 Mexico City, Mexico; Borgatta, 15700 Mexico City, Mexico; TP Orthodontics Inc., 46350-9672 La Porte, IN, USA) were selected from the same lot. Both 0.016-inch diameter round and 0.016 × 0.022-inch rectangular cross-section wires were selected for use. These dimensions were selected because they are commonly used in clinical orthodontic treatment and are well-characterized in the orthodontic literature. The wires were obtained directly from the manufacturers and are all from the same lots; these were measured and verified with a digital micrometer (#O4OO-EEP, Electric Digital Caliper Orthodontic Tip; Orthopli, Philadelphia, Penn). A random sampling of the wires was measured for each manufacturer; for the coated wires, the coating was first dissolved using methyl ethyl ketone. Therefore, profiles of the wires were measured along their respective lengths, and the averages were recorded. For the round wires, the TP labial surface coated wires were 0.01618 inches; the TP regular wires were 0.01618 inches; the Ah Kim Pech coated wires were 0.01567; the Ah Kim Pech regular wires were 0.01612; the Borgatta coated wires were 0.01527; and the Borgatta regular wires were 0.01623. For the rectangular wires, the TP labial surface coated was 0.1562 × 0.02147; the TP regular was 0. The sample size consisted of 15 wires for both the control and experimental groups. Straight pieces of Ni-Ti wire, 35 mm back ends in length, were evaluated as previously described [10] following the recommendations with minimal modifications of the ISO standard 15841 for the three-point bending test at 36°C. A Universal Testing Machine INSTRON 5567 (Norwood, MA 02062-2643, USA) was used to evaluate the load-unload-deflection characteristics of the specimens at the Dental Biomaterials Laboratory of the National Autonomous University of Mexico (UNAM), where the equipment was properly calibrated prior to testing. Each sample was subjected to a loading and unloading cycle under a cell of Newtons. The test wires were placed on a metal fixture with two-point supports at a distance of 14 mm [11, 12]. Moreover, in a pilot study, it was observed that the arch tended to separate from the distal end of the brackets due to the deformation in the central area. Therefore, to better simulate the conditions, two more brackets were attached at 8 mm distal from each placed bracket. On the surface of the metal fixture, four bicuspid passive Damon Q self-ligating brackets (Ormco Co., Orange, California, 92867 USA), slot size 0.022′′ × 0.028′′, were adhered. Although the ISO standard 15841 recommends standard brackets, we used these kinds of brackets because they have shown less friction [13]; they therefore allowed us to reduce, to a greater extent, any unfavorable effects due to friction by stainless steel ligatures and to avoid bias. To adhere the brackets without creating deflection angles, a straight steel wire 0.019′′ × 0.025′′ thick was placed through the four preadhesion brackets, and it was removed 25 min after the last bracket was placed. To obtain straight portions of nickel-titanium wire, the two back ends of the preformed arches were collected [14]. Segments of 35 mm in length were cut, and measurements were performed with a digital calibrator. For each test, a segment was slid into the bracket slot so that the bracket lids would not open during the experiments.

**Figure 1:**
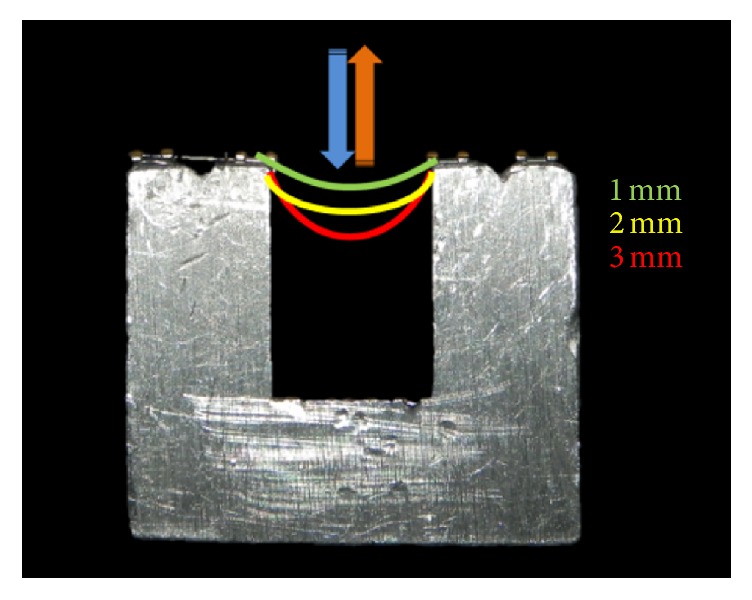
Four passive bicuspid Damon Q self-ligating brackets (Ormco Co., Orange, California, 92867, USA) were attached to the metal base, for the three-point fixture.

The test wires were secured on passive self-ligating Damon Q bicuspid brackets, slot 0.022′′ × 0.028′′ (Ormco Co., Orange, California, 92867 USA), which were attached to the surface of the metallic fixture (Figure 1). The tip of the striker was placed at the center of the sample at 7 mm from the test-wire span. The crosshead speed for loading and unloading was 5 mm/min for the round Ni-Ti wires, and the midportion of the wire was deflected. Each sample was recorded at 1, 2, 3, and 5 mm, the latter with the purpose of obtaining further information during the experiments, and the unloading values were taken at 3, 2, and 1 mm. For the rectangular wires, the crosshead speed for loading and unloading was 3 mm/min; the wire deflection was recorded at 1, 2, and 3 mm; and the unloading values were evaluated at 2 and 1 mm. For both the round Ni-Ti wires and the rectangular wires, the permanent “recovery” unload deflection was recorded at the end of each cycle. For each sample, 2 min of cycle (1 min of load and 1 min of discharge) was completed, registering the force shown on the computer screen at the end of the loading phase and the value graph issued by Calvarez MtM measurement software (Des Plaines, IL 60018, USA). For each plot, the force (*y*-axis) was obtained “by rule of three” from the final value of the load registered for 1, 2, and 3 mm of deflection (*x*-axis) of the sample. The final deformation value of the material was measured in those samples in which the traced endpoint did not coincide with the origin point (deflection 0).

All samples were tested under identical conditions. The force deflection values were registered in a Cartesian plane. The load and unload deflection span were compared between coated Ni-Ti and regular wires of the same size and from the same manufacturer, and coated Ni-Ti wires of the same dimensions were also compared among different manufacturers.

### 2.1. Statistical Analysis

Data are expressed as the mean standard error of the mean (SEM). Differences between the control and experimental load and unload deflection of the Ni-Ti regular and coated wires were determined through the variance analysis offered by Fisher's Test and the nonparametric Tukey test using GraphPad Prism 4 software (GraphPad Software Inc., San Diego, CA, USA). The level of significance was set at *p* < 0.05.

## 3. Results

### 3.1. Load and Unload Evaluation of Round 0.016′′ Ni-Ti Coated and Regular Wires

**Figure 2:**
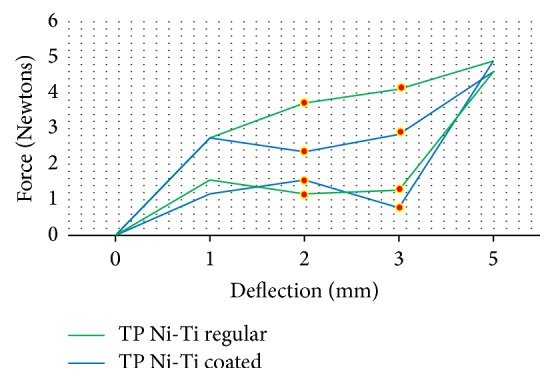
Comparison of the mean force (N) load and unload deflection between the coated and regular Ni-Ti TP round 0.016′′ wires. There was a statistically significant decrease in the coated wire at 2 mm (*p* < 0.01) and 3 mm (*p* < 0.01) load deflection; for an unload deflection at 2 mm, a decrease (*p* < 0.01) was observed, while, at 3 mm, an increase (*p* < 0.01) was found compared to regular Ni-Ti wires.

**Figure 3:**
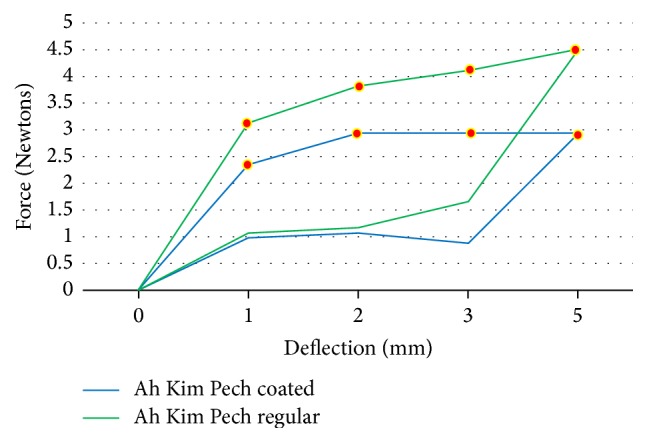
Comparison of the mean force (N) load and unload deflection between the coated and regular Ah Kim Pech 0.016′′ Ni-Ti wires. There was a statistically significant difference in the coated Ni-Ti wires at 1 mm (*p* < 0.001), 2 mm (*p* < 0.001), 3 mm (*p* < 0.001), and 5 mm (*p* < 0.001) load deflection and for unload deflection at 1 mm (*p* < 0.001), 2 mm (*p* < 0.001), and 3 mm (*p* < 0.001).

**Figure 4:**
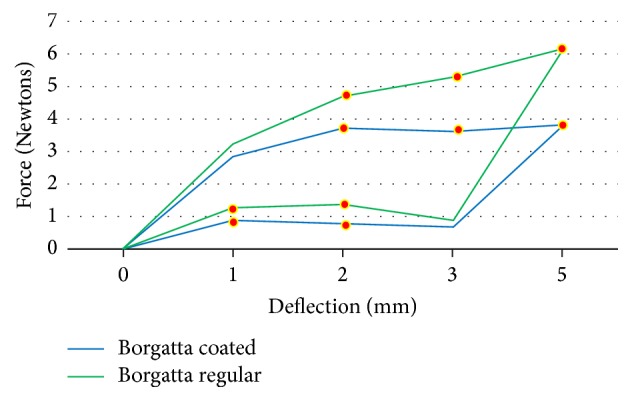
Comparison of the mean force (N) load and unload deflection between coated and regular Borgatta 0.016′′ Ni-Ti wires. There was a statistically significant decrease in the coated Ni-Ti wires at 2 mm (*p* < 0.001), 3 mm (*p* < 0.001), and 5 mm (*p* < 0.0001) load deflection compared with regular Ni-Ti wires. For the unload deflection, the coated Ni-Ti wires showed a significantly decreased deflection at 1 mm (*p* < 0.01) and 2 mm (*p* < 0.01) compared with the regular Ni-Ti wire.

The force load and unload deflections of the Ni-Ti wires were recorded in Newtons/force. The mean force load and the unload deflection values of 0.016′′ round Ni-Ti coated (*N* = 15) and regular (*N* = 15) wires are presented in Table 1. The TP-reflex coated wires showed a lower force at 2 (*p* < 0.01) and 3 mm (*p* < 0.01) load and unload deflection compared to the Ni-Ti regular wires from the same company, and the differences were significant (*p* < 0.01) (Figure 2). For the Ah Kim Pech coated wires, a lower force at 1, 2, 3, and 5 mm loading and unloading at 3 mm was observed compared to that of the regular Ni-Ti wires (*p* < 0.01) (Figure 3). Finally, compared to the control group, the Borgatta coated Ni-Ti wires showed a lower force at 2, 3, and 5 mm loading and a lower unload deflection at 1 and 2 mm (*p* < 0.01) (Figure 4).

### 3.2. Permanent Deflection of 0.016′′ Round Coated and Regular Wires

**Figure 5:**
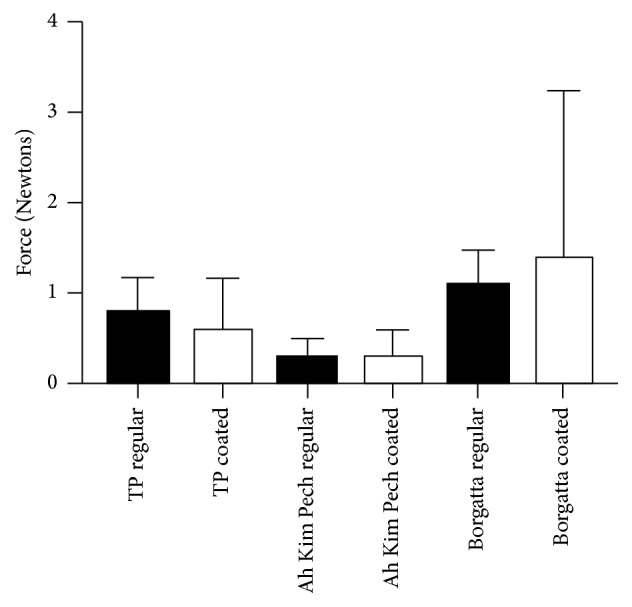
Comparison of the mean force (N) of the permanent deflection between round 0.016′′ regular and coated Ni-Ti wires. There was no statistically significant difference between the coated and regular Ni-Ti wires from the three manufacturers; nevertheless, there was a statistically significant difference between the coated TP and the Ah Kim Pech and Borgatta Ni-Ti wires (*p* < 0.01).

The permanent deflection evaluated after an unload test recovery cycle showed no significant difference when comparing the regular and coated Ni-Ti wires from the three tested manufacturers (Figure 5). Nevertheless, there was a significant trend of increased permanent deflection when the TP Ni-Ti coated wires were compared to the Ah Kim Pech product (*p* < 0.01). The Borgatta coated wire resulted in the highest permanent deflection.

Both regular and coated Borgatta archwires presented the highest values of deformation; the coated archwire had the highest standard deviation and high values of coefficient of variation, indicating that there was high variability in the deformation of the wire. The regular and coated Ah Kim Pech archwires were the least deformed (Table 2).

### 3.3. Load and Unload Evaluation of Rectangular 0.016′′ × 0.022′′ Ni-Ti Coated and Regular Wires

**Figure 6:**
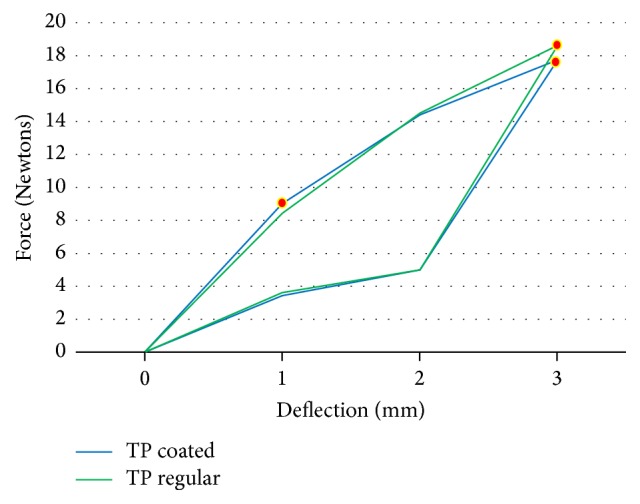
Comparison of mean force (N) load and unload deflection between the coated and regular rectangular TP Ni-Ti 0.016′′ × 0.022′′ Ni-Ti wires. There was a statistically significant increase in the coated Ni-Ti wires at 1 mm (*p* < 0.01) and a decrease at 3 mm (*p* < 0.01) load deflection compared with the regular Ni-Ti wire. There was no statistically significant difference in unload deflection.

**Figure 7:**
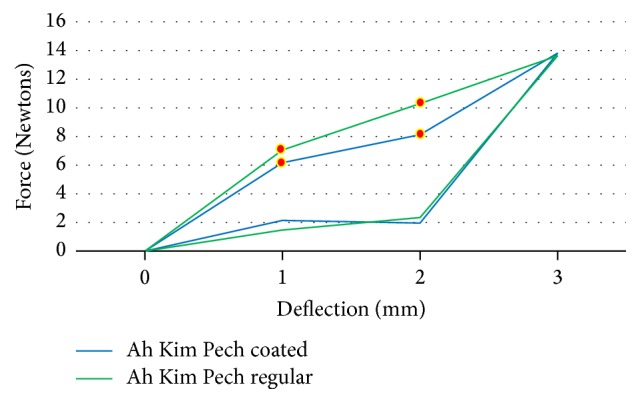
Comparison of mean force (N) load and unload deflection between the coated and regular rectangular Ah Kim Pech 0.016′′ × 0.022′′ Ni-Ti wires. There was a statistically significant decrease in the coated Ni-Ti wires at 1 mm (*p* < 0.01) and 2 mm (*p* < 0.01) load deflection compared with the regular Ni-Ti wire. There was no statistically significant difference in the unload deflection.

**Figure 8:**
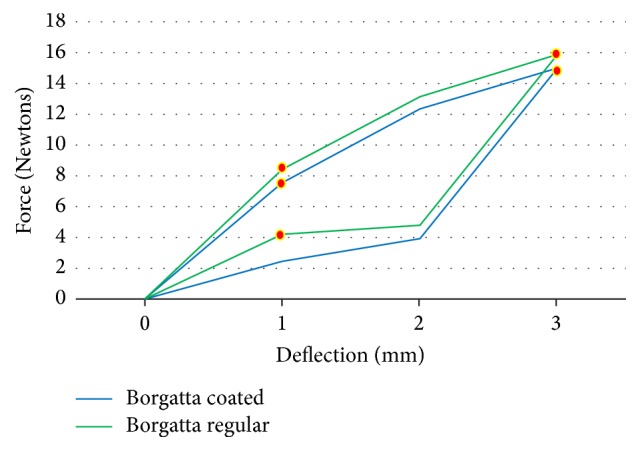
Comparison of the mean force (N) load and unload deflection between the coated and regular rectangular Borgatta 0.016′′ × 0.022′′ Ni-Ti wires. There was a statistically significant decrease in the coated Ni-Ti wires at 1 mm (*p* < 0.01) and 3 mm (*p* < 0.01) load deflection compared with the regular Ni-Ti wire. There was a statistically significant increase in the unload deflection in the regular Ni-Ti wire at 1 mm compared to that of the coated wire (*p* < 0.01).

The mean force load and the unload deflection values for rectangular 0.016′′ × 0.022′′ Ni-Ti coated and regular wires are presented in Table 3. The TP coated wires showed a significantly lower force at 1 (*p* < 0.01) and 3 (*p* < 0.01) mm loading compared to the control group. The unload deflection did not present a significant difference compared with Ni-Ti regular wires from the same company (Figure 6). For the Ah Kim Pech coated wires, a significant difference was observed, with a lower force at 1 (*p* < 0.01) and 2 (*p* < 0.01) mm loading. For the unloaded group, there was no significant difference between the coated and regular Ni-Ti wires (Figure 7). The Borgatta coated Ni-Ti wires showed a significantly lower force at 1 mm (*p* < 0.01) loading. For the unload deflection, a significant difference was observed at 1 mm (*p* < 0.001) for the regular Ni-Ti wires compared to the coated Ni-Ti wires (Figure 8).

### 3.4. Permanent Deformation of Rectangular 0.016′′ × 0.022′′ Coated and Regular Wires

**Figure 9:**
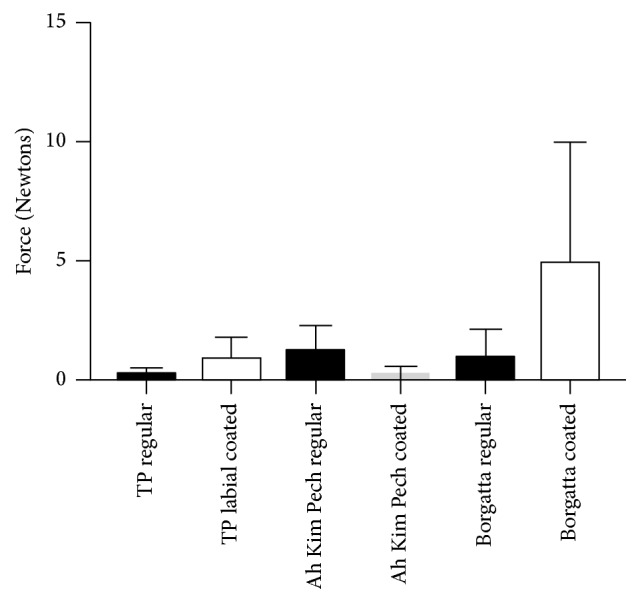
Comparison of the mean force (N) of the permanent deflection between the rectangular 0.016′′ × 0.025′′ regular and coated Ni-Ti wires. There was no statistically significant difference between the coated and regular Ni-Ti wires from TP and Ah Kim Pech; however, for the coated Borgatta wire, there was a significant increase in the permanent deflection (*p* < 0.01).

An evaluation of the permanent deformation after an unload test recovery cycle indicated no significant difference when regular and coated Ni-Ti wires were compared for the three manufacturers tested (Figure 9), except for the coated Borgatta Ni-Ti wire, which showed a significant increase. A significant difference in permanent deformation was found when the TP Ni-Ti coated wires were compared to the Borgatta wires (*p* < 0.01). Furthermore, a significant difference was observed between the deformation of the TP- Ni-Ti coated wire and that of the Borgatta wire (*p* < 0.01) (Figure 9). The Ah Kim Pech rectangular Ni-Ti coated wire had a smaller permanent recovery cycle deformation.

The Borgatta coated Ni-Ti wire segments presented greater permanent deflection, followed by the TP labial coated wire; there was less permanent deflection for the Ah Kim Pech wires. The Borgatta coated wires had the highest standard deviation. In general, the Borgatta coated wires have high coefficients of variation, particularly those with a lower average permanent deflection, because many of their values were retained at zero (median), and a few were permanently deflected.

Statistical analysis showed that there is a statistically significant difference when comparing the regular and the coated wires of the same manufacturer in all groups (Table 4).

## 4. Discussion

This study evaluated the loading, unloading, and permanent deformation of two newly available epoxy resin coated Ni-Ti archwires in comparison to a wire with a labial surface coating. The mechanical properties of these types of wire were assessed with a 3-point bending test to determine whether there is an optimum, predictable, and effective orthodontic force delivery during orthodontic mechanical treatment. Although the ISO standard 15841 supports a length of 10 mm, a deflection of −3.1 mm, head speed of 1.25 mm/min, and temperature of 36°C [15], we used a support length of 14 mm, a deflection of 3 or 5 mm, a crosshead speed of 1.25 mm/min, and a temperature of 36°C.

When epoxy resin is combined with a Ni-Ti archwire, it should ideally be combined in such a manner that the final product has the same mechanical properties as the Ni-Ti archwire without the epoxy resin, although this is difficult to achieve.

According to Senger and Ibe, the loading section of the curve represents the force required to engage the wire in the bracket, while the unloading section of the curve represents the forces applied to the teeth during the leveling and aligning phase of treatment; thus, permanent deformation of the Ni-Ti archwire must be minimized [16, 17].

Our evaluation of the new Ah Kim Pech epoxy resin coated 0.016′′ round Ni-Ti wires showed significant differences in force reduction when loading at 1, 2, 3, and 5 mm, as did the new coated Borgatta Ni-Ti wire at 3, 2, and 1 m, for unloading force deflection. Additionally, significant differences were found in the unloading force deflection between the Ah Kim Pech wire at 3 mm and the Borgatta wire at 1 and 2 mm and their controls, as reported in previous studies of epoxy resin coated Ni-Ti wires [17–19]. However, a force reduction for this type of wire was present, usually due to an inner reduction of the wire, as the epoxy resin coat increases the dimensions of the archwire [20, 21]. Nevertheless, in the inner part of these types of wires, there were no significant differences in the size, which suggests that the loading and unloading was affected by the epoxy resin cover.

In this study, self-ligating brackets were used (Damon Q bicuspid brackets, slot 0.022′′ × 0.028′′, Ormco Co., USA), as it has been demonstrated that modules and stainless steel ligatures can increase friction [19], while it is recognized that this kind of bracket reduces friction during initial alignment. Furthermore, in these experiments, four brackets were used to support the Ni-Ti wire to avoid bends in the Ni-Ti wire during the evaluation and to allow for sliding mechanics during the loading assessment [8]. Nonetheless, the loading curve presented for the round 0.016′′ Ni-Ti epoxy resin coated wires from the Ah Kim Pech and Borgatta manufacturers exhibited a lower force of deflection compared to the regular Ni-Ti wire, which is partially due to the friction between the wire and the self-ligating brackets [22], indicating a material loss of cover in the angles of the slot brackets. In contrast, in the unloading curve, a lower deflection was evident, producing a complex interaction in the loading and unloading process evaluation. This result may be influenced by the epoxy resin on the wire, which increases the dimensions of the wire [20, 21]. However, there was no significant difference in the inner part of both types of archwires, the fixation of the bracket to the base without movement, or the components of the alloy of the Ni-Ti wire [23], as this type of Ni-Ti is martensitically stable, which is a low-temperature metallurgical phase with a hexagonal structure in which shape memory can be mechanically or thermally induced. Therefore, at the end of the permanent deflection evaluation, the two types of Ni-Ti epoxy resin coated wire recovered and returned to the harder austenitic phase, although the Borgatta coated archwire had the highest permanent deflection (1.37 ± 1.86) followed by the regular Ni-Ti (1.07 ± 0.39) compared with the Ah Kim Pech coated wire (0.29 ± 0.29) and the regular Ni-Ti Archwire (0.29 ± 0.19).

To produce reference values of the mechanical properties of these new epoxy resin coated Ni-Ti wires, they were compared with the TP-reflex Ni-Ti archwires, with only a labial surface covered with epoxy resin, as this feature of the wire reduces friction. A significant difference in force reduction was found for loading at 2 and 3 mm and for unloading force deflection at 2 and 3 mm, compared with regular Ni-Ti wires. There was no permanent deformation for either the coated or regular Ni-Ti wire. The force delivered by this type of Ni-Ti wire is consistent with a previous study that reported that the force in Newtons exceeds the ideal value [8]. Considering these mechanical properties, the Borgatta wires showed a lower force, followed by the Ah Kim Pech wire compared with the TP-reflex coated Ni-Ti wires. Nevertheless, wires from the three manufacturers demonstrated optimal mechanical properties with respect to elasticity and resilience.

The mechanical properties of the rectangular 0.16′′ × 0.022′′ Ni-Ti epoxy resin wires from the Ah Kim Pech and Borgatta manufacturers and the TP-reflex rectangular Ni-Ti labial surface coated wires differed from those of round Ni-Ti wires. There was a significant force reduction at 1 and 3 mm loading, while the unloading in the TP-reflex rectangular epoxy labial resin wire remained similar to that of the regular Ni-Ti wire. There was a significant force reduction at 1 and 2 mm for the Ah Kim Pech and at 1 mm for the Borgatta Ni-Ti epoxy resin coated wires compared with the regular Ni-Ti wires, but the Ni-Ti wires from both manufacturers were unaffected in the unloading evaluation. The mechanical properties of this type of wire are consistent with those reported in previous studies [18, 19, 24], which recorded an optimal force. Among the three types of Ni-Ti-epoxy resin coated wire, the Ah Kim Pech wires showed the lowest force loading deflection. After the evaluation of the epoxy resin coated Ni-Ti archwires and using the Ni-Ti polytetrafluoroethylene with only the labial surface coated as a reference, we can state that both types of coating produced changes in the mechanical properties; these were exhibited as a reduction in the applied force. In addition, it was previously reported that the epoxy resin coated Ni-Ti archwires show severe deterioration and greater surface roughness on the surface of the archwires [25]. Therefore, the polytetrafluoroethylene labial coated surface may provide more advantageous properties than the former, although the Ah Kim Pech coated wire also had a lower permanent deflection than the regular Ni-TI archwire, and the Borgatta coated Ni-Ti wire had a higher permanent deflection than the regular one.

All of the samples were tested under identical conditions at the same temperature. A limitation of this study was that the oral environment was not simulated, and there was a lack of information on the archwires from the manufacturers.

It has been shown that the epoxy resin coated nitinol has a life of 4–6 weeks in the mouth because the oral environment could affect the epoxy resin. Nevertheless, bacterial colonization is reduced in coated wires [26], although this time could be sufficient for initial tooth alignment. Therefore, future research should focus on evaluating whether low coefficients of friction and enhanced biocompatibility are possible by modifying the surface chemistry of the polymer without affecting the resilience of the wire.

From the clinical practice perspective, it is of great value to orthodontic practice for newly available orthodontic materials offered by manufacturers to be evaluated to determine the appropriate orthodontic mechanical treatment goals for patients.

## 5. Conclusions

Within the limitations of this study, the following conclusions were drawn:

1. The coated round 0.016′′ and rectangular 0.016′′ × 0.022′′ Ni-Ti wires delivered significantly lower loading and unloading forces than regular Ni-Ti wires from the same manufacturer.
2. The permanent deformation of round 0.016′′ and rectangular 0.016 × Ni-Ti wires after an unload test recovery cycle exhibited no significant difference between regular and coated Ni-Ti wires from the same manufacturer, but there was a significant difference among wires from the three manufacturers.

## Figures and Tables

**Table 1:** Comparison of the mean force in Newtons between the regular and coated Ni-Ti round 0.016′′ wires at different deflection loads. The results are expressed as the mean ± SEM for 15 Ni-Ti wires. ^*∗*^*p* < 0.01 and ^*∗∗*^*p* < 0.001, experimental group significantly less than control.

Round 0.016′′	Loading					*p* value	Unloading					*p* value
		Mean	DS	SE	CV			Mean	DS	SE	CV	
TP Ni-Ti regular	1 mm	**2.74**	**0.19**	**2.74**	**70.80**		1 mm	**1.17**	**0.19**	**1.07**	**184.36**	
	2 mm	**3.72**	**0.29**	**3.72**	**68.15**		2 mm	**1.17**	**0.29**	**1.17**	**209.47**	^*∗*^ *p* < 0.01
	3 mm	**4.11**	**0.29**	**4.21**	**68.05**		3 mm	**0.78**	**0.29**	**0.68**	**363.14**	
	5 mm	**4.90**	**0.39**	**4.90**	**81.49**		5 mm					

TP Ni-Ti coated	1 mm	2.74	0.19	2.74	77.76		1 mm	1.17	0.19	1.27	118.85	
	2 mm	3.92	0.29	4.02	65.99	^*∗*^ *p* < 0.01	2 mm	1.56	0.19	1.66	125.72	
	3 mm	4.41	0.29	4.51	66.29	^*∗*^ *p* < 0.01	3 mm	1.27	0.19	1.27	180.44	^*∗*^ *p* < 0.01
	5 mm	4.60	0.39	4.80	78.55		5 mm					

Ah Kim Pech Ni-Ti regular	1 mm	**3.13**	**0.19**	**3.13**	**70.11**		1 mm	**1.17**	**0.19**	**1.17**	**167.10**	
	2 mm	**3.82**	**0.29**	**3.82**	**78.35**		2 mm	**1.07**	**0.19**	**1.07**	**150.72**	
	3 mm	**4.11**	**0.29**	**4.11**	**78.45**		3 mm	**1.66**	**3.13**	**0.88**	**1836.98**	
	5 mm	**4.51**	**1.47**	**4.51**	**330.87**		5 mm					

Ah Kim Pech Ni-Ti coated	1 mm	2.35	0.19	2.35	97.96	^*∗*^ *p* < 0.01	1 mm	1.07	0.19	0.98	223.39	
	2 mm	2.94	0.19	2.94	74.82	^*∗*^ *p* < 0.01	2 mm	0.10	0.19	0.98	215.05	
	3 mm	2.94	0.29	3.04	87.76	^*∗*^ *p* < 0.01	3 mm	0.88	0.19	0.88	220.84	^*∗∗*^ *p* < 0.001
	5 mm	2.94	0.29	2.94	91.59	^*∗*^ *p* < 0.01	5 mm					

Borgatta Ni-Ti regular	1 mm	**3.23**	**0.29**	**3.23**	**86.29**		1 mm	**1.27**	**0.19**	**1.27**	**182.69**	
	2 mm	**4.70**	**0.39**	**4.60**	**86.98**		2 mm	**1.37**	**0.29**	**1.37**	**222.70**	
	3 mm	**5.29**	**0.58**	**5.19**	**104.04**		3 mm	**0.88**	**0.29**	**0.98**	**263.99**	
	5 mm	**6.17**	**1.27**	**5.78**	**204.46**		5 mm					

Borgatta Ni-Ti coated	1 mm	3.04	0.58	2.94	179.16		1 mm	0.88	0.29	0.98	315.28	^*∗*^ *p* < 0.01
	2 mm	3.72	1.07	3.43	290.57	^*∗*^ *p* < 0.01	2 mm	0.78	0.29	0.78	348.33	^*∗*^ *p* < 0.01
	3 mm	3.62	0.29	3.62	87.76	^*∗*^ *p* < 0.01	3 mm	0.68	0.39	0.58	589.47	
	5 mm	3.82	0.78	3.62	187.99	^*∗∗*^ *p* < 0.001	5 mm					

**Table 2:** Comparisons of the mean force (N) of the permanent deflection between the coated and regular Nil-Ti wires by manufacturer.

0.016′′ round Ni-Ti	Mean	SD	SE	CV
TP regular	0.78	0.39	0.98	427.27
TP coated	0.58	0.58	0.58	1008.02
Ah Kim Pech regular	0.29	0.19	0.29	616.44
Ah Kim Pech coated	0.29	0.29	0.29	860.14
Borgatta regular	1.07	0.39	1.27	395.99
Borgatta coated	1.37	1.86	0.68	1342.92

**Table 3:** Comparison of mean force in Newtons between the regular and coated Ni-Ti rectangular 0.016′′ ×  0.022′′ wires at different deflection loads. The results are expressed as the mean ± SEM for 15 Ni-Ti wires. ^*∗*^*p* < 0.01, experimental group significantly less than control.

0.016′′ × 0.022′′ Ni-Ti	Loading					*p* value	Unloading					*p* value
		Mean	DS	SE	CV			Mean	DS	SE	CV	
TP Ni-Ti regular	1 mm	8.43	0.39	8.43	43.05	^*∗*^ *p* < 0.01	1 mm	3.62	0.29	3.62	66.97	
	2 mm	18.73	0.68	18.63	36.48		2 mm	5.00	0.29	5.00	51.77	
	3 mm	18.73	0.68	18.63	36.48		3 mm					

TP Ni-Ti coated	1 mm	**9.02**	**0.88**	**9.12**	**92.96**		1 mm	**3.43**	**0.68**	**3.62**	**191.32**	
	2 mm	**17.75**	**1.37**	**17.65**	**77.27**		2 mm	**5.00**	**1.27**	**5.68**	**249.67**	
	3 mm	**17.75**	**1.37**	**17.65**	**77.27**	^*∗*^ *p* < 0.01	3 mm					

Ah Kim Pech Ni-Ti regular	1 mm	7.06	0.39	7.15	51.68		1 mm	1.47	0.29	1.56	184.65	
	2 mm	13.63	1.17	13.72	83.55		2 mm	2.35	0.39	2.45	147.68	
	3 mm	13.63	1.17	13.72	83.55		3 mm					

Ah Kim Pech Ni-Ti coated	1 mm	**6.17**	**0.58**	**6.27**	**91.59**	^*∗*^ *p* < 0.01	1 mm	**2.15**	**1.86**	**1.76**	**857.00**	
	2 mm	**13.82**	**2.15**	**14.02**	**156.51**	^*∗*^ *p* < 0.01	2 mm	**1.96**	**2.15**	**1.37**	**1105.20**	
	3 mm	**13.82**	**2.15**	**14.02**	**13.82**		3 mm					

Borgatta Ni-Ti regular	1 mm	8.43	0.39	8.33	40.59		1 mm	4.21	1.47	3.92	337.93	^*∗*^ *p* < 0.01
	2 mm	15.88	1.07	15.69	67.17		2 mm	4.80	0.98	5.00	210.64	
	3 mm	15.88	1.07	15.69	67.17		3 mm					

Borgatta Ni-Ti coated	1 mm	**7.55**	**1.27**	**7.74**	**166.12**	^*∗*^ *p* < 0.01	1 mm	**2.45**	**1.17**	**2.45**	**464.34**	
	2 mm	**15.00**	**2.25**	**15.39**	**149.35**		2 mm	**3.92**	**1.47**	**4.11**	**377.16**	
	3 mm	**15.00**	**2.25**	**15.39**	**149.35**		3 mm					

**Table 4:** Comparisons of the mean force (N) of the permanent deflection between the coated and regular Ni-Ti wires by manufacturer.

	Mean	DS	SE	CV
TP regular	0.19	0.29	0.00	1796.67
TP coated	0.88	0.88	0.98	988.90
Ah Kim Pech regular	1.17	1.07	0.78	930.74
Ah Kim Pech coated	0.19	0.39	0.00	1563.96
Borgatta regular	0.88	1.27	0.78	1377.73
Borgatta coated	4.90	5.00	3.33	992.62
